# In and out of the mitochondrial intermembrane space

**DOI:** 10.1002/pro.70493

**Published:** 2026-02-12

**Authors:** Fara van der Schans, Kostas Tokatlidis, Daniela G. Vitali

**Affiliations:** ^1^ School of Molecular Biosciences University of Glasgow Glasgow UK

**Keywords:** intermembrane space, Mia40, oxidative folding, proteostasis

## Abstract

Mitochondria are essential organelles constituted by two membranes, the outer (OMM) and inner mitochondrial membrane (IMM), and two aqueous compartments, the intermembrane space (IMS) and the matrix. Although mitochondria contain their own genome, which encodes for 13 proteins in humans (8 in budding yeast), the vast majority (99%) of mitochondrial proteins are encoded by the nuclear DNA and imported into the organelle co‐ or post‐translationally. The IMS lies between the cytosol and the matrix, making it a strategic hub for monitoring the mitochondrial proteome. All IMS‐resident proteins are nuclear‐encoded and play critical roles in cellular pathways, such as redox regulation, calcium signaling, apoptosis, and hypoxia response. Furthermore, most mitochondrial proteins pass through the IMS en route to their final destination within the organelle. During this transit, their targeting and folding states are carefully monitored: properly folded proteins are retained, while misfolded or potentially toxic polypeptides are retrotranslocated and degraded. In this review, we highlight the mechanisms by which proteins are sorted into the IMS and discuss its central role in regulating mitochondrial proteostasis and maintaining mitochondrial function.

## 1 THE MITOCHONDRIAL INTERMEMBRANE SPACE PROTEOME

The mitochondrial proteome is regulated by the coordinated expression of nuclear and mitochondrial genomes. The latter encodes 13 genes, meaning over 99% of mitochondrial proteins (~1500 in humans or ~1100 in budding yeast) must be co‐ or post‐translationally targeted to mitochondria. The intermembrane space (IMS) is the most constricted mitochondrial subcompartment and is uniquely positioned between the mitochondrial matrix and cytosol (Wiedemann and Pfanner 2017). As such, the IMS acts as a sensitive logistics hub that can integrate cellular signals to fine‐tune the mitochondrial proteome accordingly (Habich et al. 2019b).

The IMS proteome consists of approximately 150 soluble and membrane‐anchored proteins with IMS‐exposed domains, and this list continues to grow (Erdogan and Riemer 2017; Hung et al. 2014; Longen et al. 2009; Modjtahedi et al. 2016; Morgenstern et al. 2021; Rath et al. 2021; Vögtle et al. 2012). In contrast to matrix‐targeted proteins, which tend to import via one common pathway, import into the IMS occurs through several mechanisms including the mitochondrial intermembrane space assembly (MIA) import pathway, stop‐transfer pathway, and other non‐canonical mechanisms (Edwards et al. 2021).

## 2 MIA IMPORT PATHWAY

The best characterized import mechanism into the IMS is the MIA import pathway. Also referred to as the disulfide relay system or oxidative folding pathway, this mode of import mediates the biogenesis of about a third (~50 proteins) of IMS proteins. Substrates that utilize the MIA pathway are diverse in structure and function and have various roles in redox regulation, anti‐oxidant responses, biogenesis of the electron transport chain, iron–sulfur cluster biosynthesis, and mitochondrial dynamics (Zarges and Riemer 2024).

The two main players in this pathway are the oxidoreductase CHCHD4/MIA40 (Mia40 in yeast) and the sulfhydryl oxidase ALR/GFER (Erv1 in yeast), which are both essential genes and are highly conserved from yeast to humans (Habich et al. 2019b). The essentiality of the MIA pathway is further underlined by in vivo studies, where knocking out *Mia40* or *Alr* is lethal in yeast (Chacinska et al. 2004), mice (Hangen et al. 2015), and zebrafish (Sokol et al. 2018).

### 2.1 Essential components of the MIA pathway

MIA40 is structurally characterized by a coiled‐coil‐helix‐coiled‐coil‐helix (CHCH) domain that contains the functionally important redox‐active cysteine‐proline‐cysteine (CPC) motif, adjacent to a hydrophobic substrate‐binding groove. This hydrophobic cleft recognizes specific internal substrate sequences known as mitochondrial IMS‐targeting signals (ITS) (also named IMS‐sorting signals or MISS) (Banci et al. 2010; Koch and Schmid 2014; Milenkovic et al. 2009; Sideris et al. 2009). This type of IMS‐specific targeting signal consists of only nine amino acid residues and is sufficient for crossing the outer mitochondrial membrane (OMM) and for targeting nonmitochondrial proteins (Milenkovic et al. 2009; Sideris et al. 2009). It can form an amphipathic helix with crucial hydrophobic residues on the side of the docking cysteine and dispensable charged residues on the other side. Structurally it complements the substrate cleft of Mia40 via hydrophobic interactions. Alanine scanning and cysteine mutagenesis have identified a consensus motif of X[Ar]XX[Hy][Hy]XXC, with the aromatic (Ar) and hydrophobic (Hy) amino acids in positions −7, −4, and −3 compared to the docking cysteine being the most critical ones. The important function of the ITS/MISS is to prime a unique cysteine (at the N‐ or C‐terminal side of the ITS/MISS) for docking to Mia40 (Sideris et al. 2009).

There are some key structural differences between yeast and human MIA40, although their functional core domain is highly conserved (Hofmann et al. 2005). Human MIA40 is a small soluble protein (16 kDa) and is localized to the mitochondrial inner membrane (IMM) through an NAD(H)‐regulated interaction with the IMM tethered apoptosis inducing factor 1 (AIF) protein, thereby linking mitochondrial import and metabolism (Brosey et al. 2025; Hangen et al. 2015). In comparison, yeast Mia40 is much larger (40 kDa) as it contains an N‐terminal transmembrane region that tethers it to the IMM (Kawano et al. 2009).

The FAD‐linked sulfhydryl oxidase ALR is the second essential component for oxidative folding. ALR has high sequence conservation and homology with its yeast homolog Erv1 (Gandhi 2012); however, some key differences do exist. In yeast, Erv1 organizes into homodimers stabilized by hydrophobic interactions (Bien et al. 2010), whereas human ALR stabilizes the dimerization between its N‐terminal shuttle domain and C‐terminal FAD‐binding core domain via two additional disulfide bonds. This configuration results in the coordination of the FAD factor in its hydrophobic pocket (Daithankar et al. 2010; Wu et al. 2003).

Altogether, the fundamental process of oxidative folding is well conserved between yeast and humans, although recent proteomic and genetic studies reveal that mammalian oxidative folding has an expanded repertoire of non‐canonical substrates (Balasco et al. 2025), requires additional auxiliary factors (such as AIF) (Meyer et al. 2015), and is additionally influenced by cellular context (such as hypoxia and calcium signaling) (Petrungaro et al. 2015; Yang et al. 2012).

### 2.2 MIA40‐dependent oxidative folding mechanism

**FIGURE 1:**
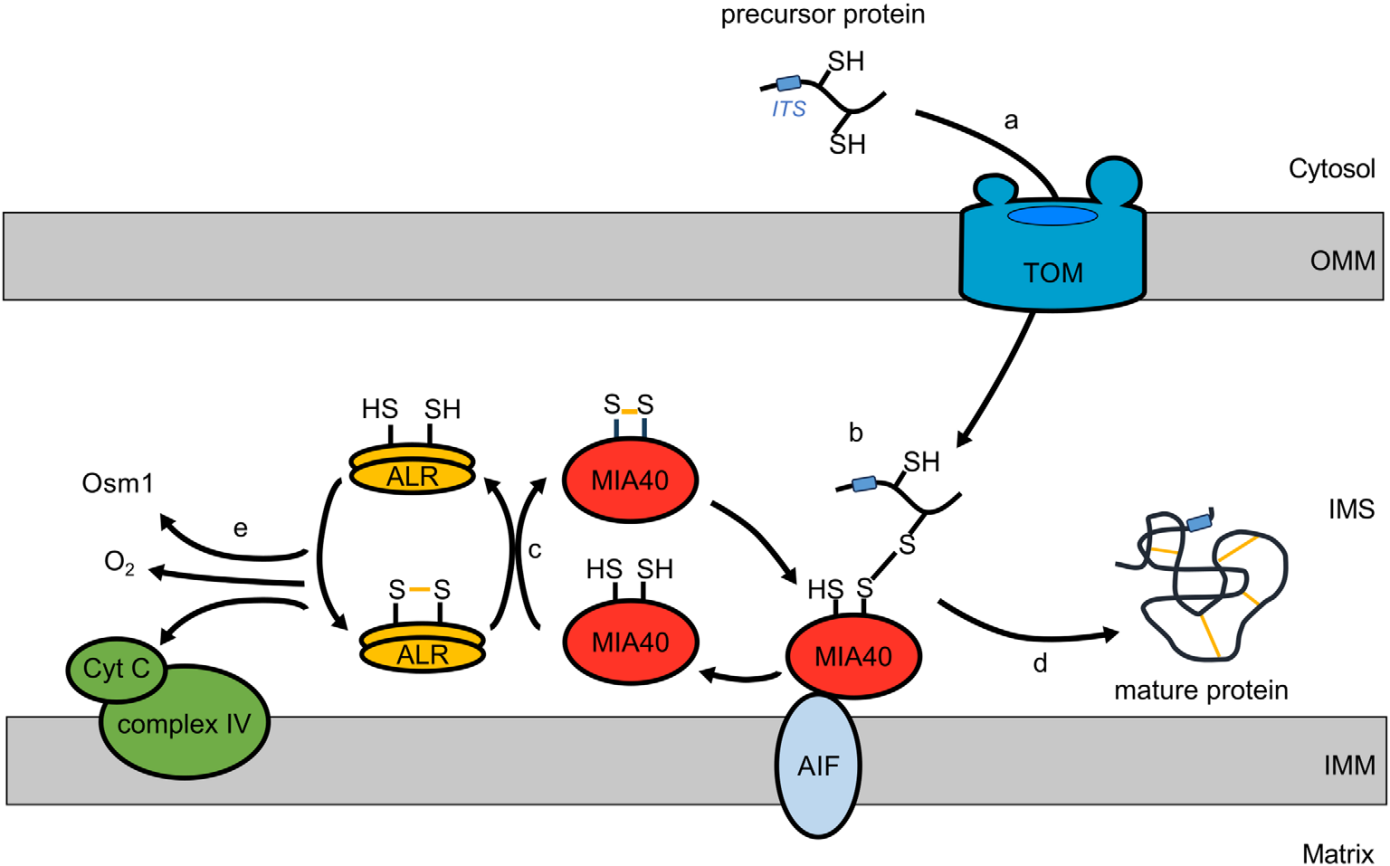
MIA import pathway into the IMS. (a) MIA pathway precursor proteins are small, cysteine‐rich substrates that initially cross the translocase of the outer membrane (TOM) in a reduced, unfolded state. (b) Once in the IMS, the hydrophobic cleft of MIA40 recognizes the substrate's internal targeting signal (ITS) through non‐covalent interactions in the “sliding” step. In the “docking” step, the catalytic CPC motif of MIA40 forms a transient intermolecular disulfide bond. (c) Subsequent electron transfer from MIA40 to the sulfhydryl oxidase ALR releases the folded, mature precursor protein into the IMS. (d) ALR then passes its electrons to a terminal electron acceptor such as cytochrome c, complex IV of the electron transport chain, and molecular oxygen to generate hydrogen peroxide, or other acceptors under anaerobic conditions, such as Osm1 in yeast. Note, in the human system MIA40 is localized to the inner membrane through an NAD(H)‐regulated interaction with apoptosis inducing factor 1 (AIF).

MIA substrates initially cross the OMM via the translocon of the outer membrane (TOM) complex in a reduced and unfolded state (Edwards et al. 2021) (Figure 1a). In general, MIA substrates only require the Tom40 channel and accessory protein Tom5 for initial translocation, thus operating independently from other major TOM receptors (Araiso et al. 2019; Gornicka et al. 2014). However, recent evidence suggests that the TOM20 receptor supports the efficient import of specific human MIA substrates, such as MIC19 and CHCHD6 (Marada et al. 2024). Together, this highlights that the exact requirements for IMS import may be species‐ and substrate‐specific but also reflects the regulatory complexity of the human mitochondrial proteome.

Upon entry into the IMS, the hydrophobic substrate‐binding cleft of MIA40 recognizes the hydrophobic ITS/MISS sequence of the incoming substrate and positions the substrate near the redox‐active CPC motif of MIA40 (MIA40^CPC^) (Sideris et al. 2009). This initial non‐covalent binding step characterizes MIA40's holdase activity and is known as the “sliding” step. In the subsequent “docking” step, the oxidoreductase function of MIA40 catalyzes the transient intermolecular disulfide bond between the substrate and MIA40^CPC^ (Figure 1b). MIA40 then transfers two electrons to the FAD‐linked ALR (Figure 1c). As a result of the interaction with Mia40, the precursor protein is released into the IMS in a stable and folded state (Figure 1d), and in the process, re‐oxidized MIA40 undergoes further oxidative folding cycles (Banci et al. 2009; Peleh et al. 2016). Lastly, further internal electron transfers within ALR to its FAD flavin moiety (which can transfer both 1‐electron and 2‐electrons) are followed by electron transfer to the mobile 1‐electron carrier cytochrome c, which passes its electrons to the cytochrome oxidase (COX or complex IV) complex of the respiratory chain and finally to molecular oxygen (Figure 1e) (Dabir et al. 2007). Moreover, the MIA pathway can operate in anaerobic conditions. In yeast, Osm1 accepts the final electron from Erv1 thereby bypassing the need for oxygen (Figure 1e) (Neal et al. 2017); however, other unidentified electron acceptors or small molecules may also be involved. No definitive alternative electron acceptor has been identified for the human MIA pathway under anaerobic or hypoxic conditions.

### 2.3 Importance of redox balance in the IMS

The IMS is a comparatively oxidizing cellular compartment that ensures efficient protein sorting and folding. However, reactive oxygen species (ROS) are a common by‐product in the IMS due to respiratory chain activity and oxidative folding (Habich et al. 2019b). Therefore, maintaining this redox balance is crucial, as excessive oxidative stress leads to the damage of proteins, lipids, and DNA, thereby compromising cellular homeostasis and mitochondrial function (Habich et al. 2019b). In yeast, the most well‐characterized detoxifying system is a cytochrome c peroxidase (Ccp1)‐cytochrome c recycling system (Dabir et al. 2007). In addition, glutathione peroxidase 3 (Gpx3) is targeted to the IMS under oxidative stress following alternative translation from a non‐AUG codon (Kritsiligkou et al. 2017), where it may also have an anti‐oxidant role. Reducing systems are equally important for redox balance. For instance, thioredoxin 1 (Trx1) and thioredoxin reductase 1 (TrR1) have also been identified in the yeast IMS proteome (Vögtle et al. 2012), although their functional role remains unclear. Presumably, the thioredoxin system helps scavenge ROS by reducing oxidized peroxiredoxin proteins in an NADPH‐dependent manner (Cardenas‐Rodriguez et al. 2021). In humans, several putative hydrogen peroxide detoxification mechanisms have been identified in the IMS, most notably peroxiredoxins (PRDX3 and PRDX4) and glutathione peroxidase 4 (GPX4) (Hung et al. 2014); however, their functional role in this process also remains unclear.

## 3 PRECURSOR PROTEINS OF THE IMS

Classic precursor proteins of the MIA pathway are small and have a highly conserved twin cysteine motif (CX_N_C), where cysteines are typically separated by three (CX_3_C) or nine (CX_9_C) amino acid residues (Balasco et al. 2025).

### 3.1 Classic MIA substrates

**FIGURE 2:**
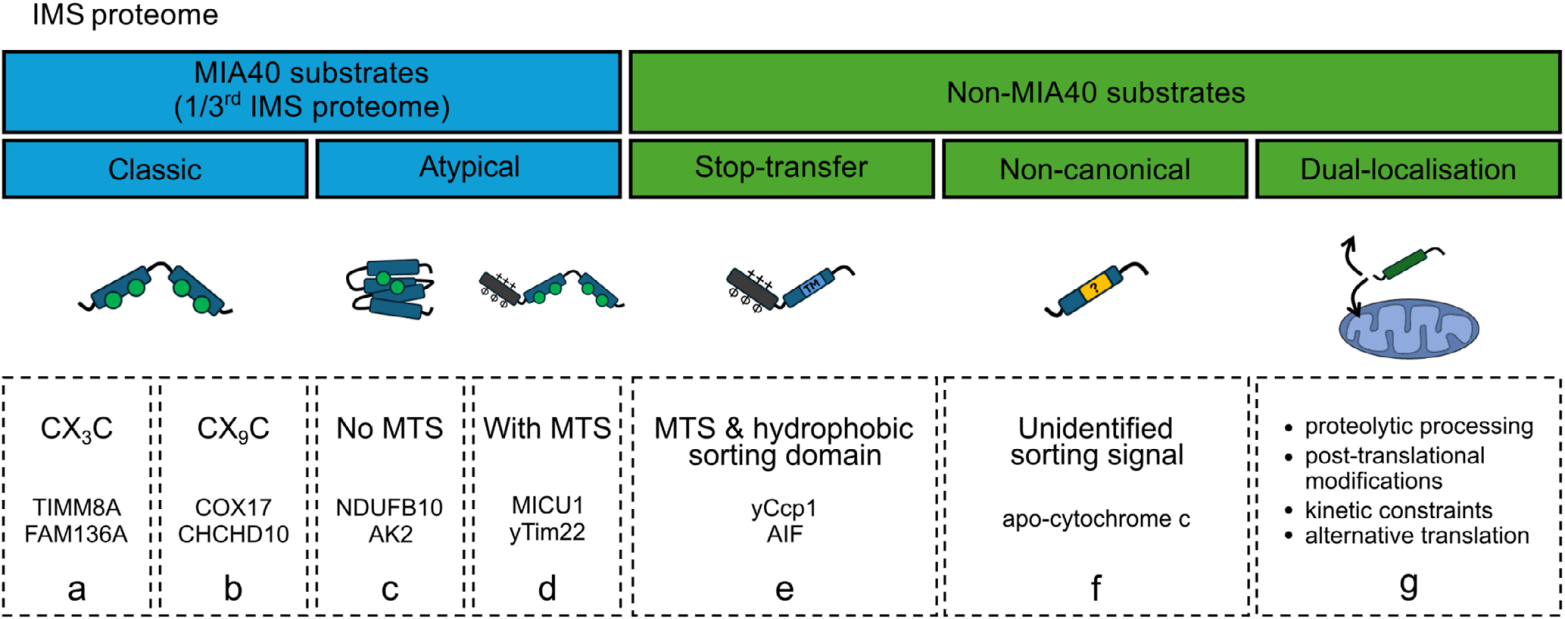
Classes of the IMS substrates. The IMS proteome consists of approximately 150 soluble and membrane‐anchored proteins with IMS‐exposed domains. Broadly these can be divided into MIA40‐dependent and ‐independent substrates. Roughly a third of the IMS proteome are MIA pathway substrates and either have a classic CX_3_C (a) or CX_9_C (b) motif or an atypical and complex cysteine motif (c, d). The latter can be further subdivided into MIA substrates without (c) or with (d) a mitochondrial targeting sequence (MTS). Overall, this highlights the incredible capacity of MIA40 to accommodate such a diverse array of topologies (e). Alternatively, resident IMS proteins are imported via a stop‐transfer mechanism via a cleavable N‐terminal MTS and internal hydrophobic sorting signal (f). Some IMS proteins lack a clear targeting signal altogether and import via non‐canonical pathways (g). In addition, a subset of IMS proteins is dually localized, a process mediated by various mechanisms that plays an important role in maintaining cellular homeostasis.

MIA substrates with a twin CX_3_C motif (Figure 2a) represent the family of small TIMM proteins and are IMS‐localized chaperones that organize into soluble hetero‐hexameric complexes critical for the efficient transport of hydrophobic membrane proteins across the aqueous IMS to either the IMM or the OMM (Koehler 2004). A recent addition to this family was FAM136A, which contains two twin CX_3_C motifs and interacts with MIA40 during import. Structurally, it is similar to small TIMM proteins and likewise prevents the aggregation of IMS proteins (Zarges et al. 2025).

Twin CX_9_C motif containing MIA substrates (Figure 2b) have diverse roles in respiratory chain biogenesis, lipid homeostasis, and mitochondrial dynamics and can broadly be classified into functional families (Balasco et al. 2025). The CHCH domain‐containing family of proteins is functionally diverse and plays roles in mitochondrial cristae integrity (CHCHD3/Mic19, CHCHD6/Mic19, CHCHD10/Mic17) (An et al. 2012; Darshi et al. 2012; Lehmer et al. 2018), mitochondrial ribosome biogenesis (CHCHD1/Mrp10) (Longen et al. 2014), complex IV‐copper chaperone activity (CHCHD7/Cox23) (Banci et al. 2012), and in driving MIA40‐dependent import, since CHCHD4/MIA40 itself is a classical substrate of the MIA pathway (Modjtahedi et al. 2016). NADH:ubiquinone oxidoreductase family (NDUF) proteins also belong to this class of MIA substrate and consist of several accessory and structural subunits of respiratory chain complex I (CI) (Habich et al. 2019a). Four out of the 14 evolutionarily conserved core subunits of CI are in the IMS (Stroud et al. 2016). Three of these (NDUFA8, NDUFS5, and NDUFB7) are typical twin CX_9_C motif MIA substrates (Szklarczyk et al. 2011), whereas NDUFB10 relies on MIA for import but contains two extra disulfide bonds to stabilize the complex (Friederich et al. 2017). Lacking any of these four CI subunits is detrimental to CI assembly (Stroud et al. 2016). Furthermore, a eukaryotic genome‐wide analysis found that six of the twin CX_9_C proteins were members of the COX family (Gladyck et al. 2021). Similarly, COX‐related membrane component proteins, important for COX assembly, also contain CX_9_C motifs (Balasco et al. 2025).

### 3.2 Atypical MIA substrates with complex cysteines

Some MIA40 substrates have atypical or complex cysteine motifs (Figure 2c) which highlight the remarkable diversity of MIA40 and its capacity to accommodate diverse protein topologies. A well‐characterized example of this in yeast is the copper chaperone Ccs1 which has two atypical cysteines (CX_36_C) and becomes oxidized during import. Additionally, the presence of Ccs1 in the IMS helps regulate the levels of the Cu‐Zn superoxide dismutase Sod1 in the IMS (Klöppel et al. 2011). Notable examples observed in humans are MIC19, which is important for the mitochondrial contact site and cristae organizing system (MICOS) and has a CX_10_C motif (Ueda et al. 2019), and C9orf72, important for energy homeostasis, with a CX_2_C‐X_29_‐CX_3_C motif (Wang et al. 2021). Similarly, adenylate kinase 2 (AK2) has an odd number of conserved cysteines (C40, C42, and C92) with unconventional spacing (Finger et al. 2020). Moreover, NDUFB10, an accessory protein of CI, has an unusual cysteine motif (CX_6_C/CX_11_C) (Friederich et al. 2017), as do members of the COX assembly protein family (Balasco et al. 2025).

### 3.3 MTS containing MIA substrates

Another atypical class of MIA substrates is with an N‐terminal mitochondrial targeting sequence (MTS) (Figure 2d). These include the regulatory protein MICU1 of the mitochondrial calcium uniporter (Petrungaro et al. 2015), yeast Tim22, which has an atypical CX_98_C motif (Wrobel et al. 2013), and the IMS metalloprotease Atp23 (Weckbecker et al. 2012). A unique example of this class of substrate is HCLS1‐associated protein X‐1 (HAX‐1), which has a weak N‐terminal MTS that drives translocation. Interestingly, HAX‐1 lacks cysteines entirely and thus the holdase activity of MIA40 provides stability to HAX‐1 post‐import to prevent its aggregation and degradation by IMS proteases (Rothemann et al. 2024). Altogether, these substrates are imported in a membrane‐potential dependent manner and are trapped in the IMS due to MIA40 activity.

### 3.4 Other import mechanisms into the IMS

#### 3.4.1 Stop‐transfer import

Nuclear‐encoded proteins destined for the IMS are also targeted via the stop‐transfer pathway (Figure 2e). This substrate class contains a bipartite presequence containing two functional components: a cleavable N‐terminal MTS and an internal hydrophobic region known as the “stop‐transfer” sorting domain. During stop‐transfer import the bipartite substrate is threaded through the TOM channel and is partially translocated through the TIM23 channel in a membrane‐potential dependent manner (Kizmaz et al. 2024). Consequently, the hydrophobic transmembrane region, or “stop‐transfer” domain, halts import in TIM23, and the substrate is laterally released into the IMM via a lateral gate of the TIM23 channel. Recent biochemical and structural analyses highlight the central role that the TIM17 subunit, a core component of the TIM23 translocase, plays as a protein‐conducting half‐channel to facilitate lateral preprotein release (Fielden et al. 2023; Sim et al. 2023; Zhou et al. 2023). This is followed by two cleavage events: firstly, the matrix processing peptidase (MPP) cleaves the presequence on the matrix side and secondly, the IMS proteases IMP1/2 or PARL cleave the protein on the IMS side, leading to the release of the mature protein into the IMS (Saita et al. 2017, 2018; Vögtle et al. 2012).

Classic IMS substrates of the stop‐transfer pathway include cytochrome b2 and cytochrome c1, which were first characterized in yeast almost 40 years ago (Glick et al. 1992). Other examples in yeast include GTPase Mgm1 (Herlan et al. 2004) and Ccp1 (Michaelis et al. 2005), both of which require cleavage events for maturation. Mammalian studies have also identified a specific subset of pro‐apoptotic proteins including AIF (Susin et al. 1999), endonuclease G (Ohsato et al. 2002), and Smac/DIABLO (Saita et al. 2017) as bipartite presequence containing substrates that localize to the IMS.

#### 3.4.2 Unknown or unconventional import into the IMS

Some IMS proteins do not have a stop‐transfer sequence or require MIA40 for their import, suggesting there are unconventional import pathways to the IMS (Figure 2f). Although detailed mechanisms of this are limited, notable examples are apo‐cytochrome c and cytochrome c haem lyase (CCHL). For instance, the apo‐cytochrome c protein is imported into the IMS in a Tim22‐dependent manner and is haem‐loaded by CCHL, which folds cytochrome c and retains it in the IMS (Diekert et al. 2001). Nonetheless, the exact driving force for their import remains unclear since neither protein requires ATP hydrolysis or an IMM potential (Edwards et al. 2021).

### 3.5 Dually localized IMS proteins

There is also a growing subset of IMS proteins that display dual‐localization with other cellular compartments (Figure 2g), which may arise through proteolytic processing, post‐translational modifications, kinetic constraints, and alternative translation (Pines et al. 2024).

Perhaps the best characterized dual sorting mechanism is proteolytic processing. Two recent examples of this are peroxiredoxin 3 (Prdx3) and the lipid‐transfer protein STARD7. The dual localization of Prdx3 between the mitochondrial matrix and IMS is regulated by competing proteolytic processing events. For matrix import, the N‐terminal MTS of Prdx3 is processed by two sequential cleavage steps, whereas IMS import is achieved via cleavage by the inner membrane peptidase (IMP), although the exact cleavage site for IMS sorting remains unknown (Gomes et al. 2024). Similarly, the dual sorting of STARD7 between the IMS and cytosol depends on proteolytic cleavage via either matrix MPP peptidase or rhomboid protease PARL on the IMM leaflet facing the IMS (Deshwal et al. 2023).

Protein sorting can also be driven by alternative translation. For instance, oxidative stress results in the N‐terminally extended Gpx3 protein translocating from the cytosol to the mitochondrial IMS in yeast (Kritsiligkou et al. 2017). Likewise, the dual sorting of Osm1 to the ER follows translation initiation from the first ATG codon, whereas the second in‐frame ATG codon generates a shorter isoform that is targeted to the mitochondrial IMS (Neal et al. 2017).

In some cases, specific folding requirements and kinetic import rates drive precursor targeting (Pines et al. 2024). In the cytosol, the TP53‐regulated inhibitor of apoptosis (TRIAP1) preferentially adopts a non‐native disulfide kinetic trap and exists in a metastable, molten globule state with exposed hydrophobic patches that are protected by cytosolic HSP70. However, targeting to the IMS and the presence of MIA40 accelerates the folding rate by 30‐fold, thereby overcoming this kinetic trap and selectively driving the formation of native disulfide bonds. In the IMS, TRIAP1 helps regulate lipid homeostasis by mediating phospholipid trafficking between mitochondrial membranes to sustain mitochondrial function (Pujols et al. 2025). Similarly, the import of adenylate kinase (Adk1), a phosphotransferase with roles in oxidative metabolism in yeast, is governed by slow‐folding kinetics (similarly to Ccs1), which allows for slow folding in the cytosol and rapid folding and trapping in the IMS (Angermayr et al. 2001).

Furthermore, post‐translational modifications can also influence import capacity and localization. This was observed for the yeast nucleoside diphosphate kinase (Ynk1), which is dually localized between the cytosol and the IMS, and must be unphosphorylated and unfolded to translocate across the TOM channel (Amutha and Pain 2003), presumably as the negatively charged phosphate group interferes with the acidic patches of Tom40, thereby preventing import (Tucker and Park 2019).

Then, there are some dually localized IMS proteins where the exact molecular mechanism remains elusive, such is the case of anamorsin, an essential iron–sulfur protein that is dually localized between the IMS and cytosol (Banci et al. 2011) and altered inheritance of mitochondria 32 (Aim32), a thioredoxin‐like iron–sulfur cluster ferredoxin protein that is dually localized between the IMS and matrix (Zhang et al. 2021).

Overall, dual sorting between the IMS and other cellular compartments is mediated through a variety of mechanisms and enables dynamic adaptation to metabolic demands and stress to maintain cellular homeostasis.

## 4 PROTEIN QUALITY CONTROL IN THE IMS

The coordination of the biogenesis of nuclear‐ and mitochondria‐encoded proteins and their sub‐mitochondrial targeting is challenging and exposes the organelle to a high risk of protein mistargeting or stoichiometric imbalance. Additionally, mutations, misfolding, and permanent damage caused by ROS can impair the mitochondrial proteome and threaten organelle functionality.

Accumulation of dysfunctional mitochondria affects mainly cells with high energy demand, such as neurons or cardiac cells, causing neurodegenerative or cardiovascular diseases. Moreover, altered metabolism as a consequence of dysfunctional mitochondria is associated with cancer and aging (Li et al. 2020). Therefore, a mitochondrial quality control (QC) system is essential to ensure the optimal activity of this organelle, removing any potentially dangerous or dysfunctional component. A variety of mitochondrial QC mechanisms have been discovered in recent years, acting at different levels, from the bulk removal of portions of the mitochondrial network to degradation of damaged proteins or stalled precursor polypeptides (Pfanner et al. 2025). Specifically, mitochondrial protein QC is crucial to allow the elimination of potentially toxic proteins and to rewire the organelle proteome in response to metabolic alterations.

Being at the interphase between cytosol and mitochondrial matrix, the IMS has a central position to control what gets in and what goes out of the organelle. Multiple QC mechanisms regulate not only the IMS proteome but also patrol substrates that transit in the IMS on their way to their final location.

### 4.1 Regulation of IMS proteome

IMS resident proteins play a crucial role in calcium signaling, apoptosis regulation, and hypoxia response. Additionally, as described above, they are essential for redox regulation and mitochondrial protein biogenesis, being not only involved in the oxidative folding and retention pathway, but also required for the insertion of proteins in the OMM or the IMM. Therefore, the IMS proteome must be surveyed by multiple QC mechanisms to remove potentially toxic polypeptides (e.g., misfolded, aggregated, or metastable intermediates) and ensure the organelle functionality.

#### 4.1.1 Reaching the IMS

IMS resident proteins are nuclear‐encoded, usually do not have a MTS and are imported post‐translationally in an ATP‐independent manner (Zarges and Riemer 2024). They are kept in an unfolded, import‐competent state by cytosolic chaperones to ensure efficient import into mitochondria. However, their import is slow and can cause accumulation of precursors in the cytosol, increasing the risk of premature (mis)folding, aggregation, and proteotoxic stress (Zarges and Riemer 2024).

**FIGURE 3:**
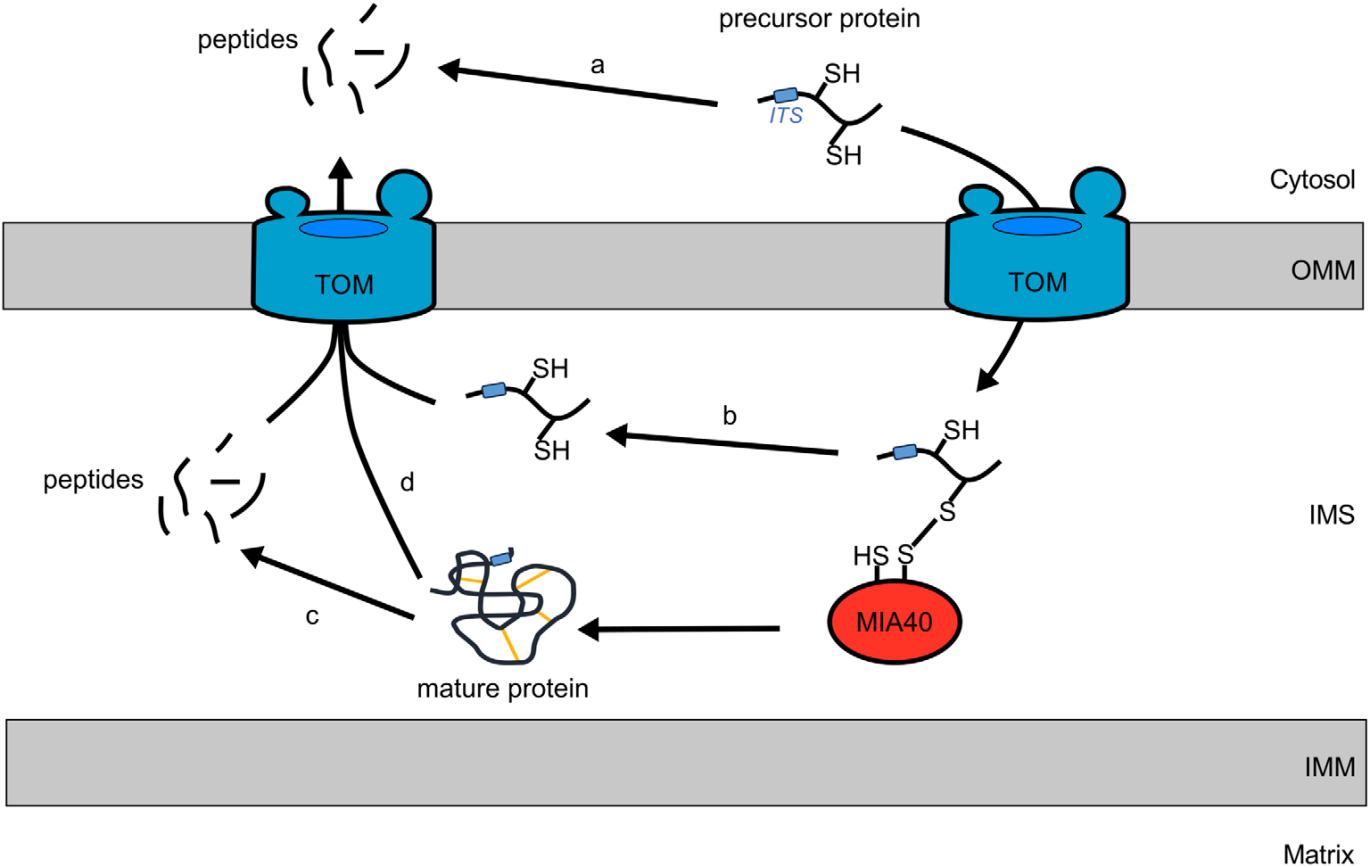
QC mechanisms monitoring IMS proteins. Proteins are subjected to QC surveillance at different stages during their journey through the mitochondria. (a) Precursor proteins accumulating in the cytosol are degraded by the ubiquitin‐proteasome system (UPS). (b) During the translocation into the IMS, disulfide relay substrates can potentially generate unproductive Mia40‐substrate intermediates, which are resolved by reducing machineries. The released substrates are then retrotranslocated through the TOM complex into the cytosol, where they will be degraded by the UPS. (c) IMS proteases monitor protein folding and degrade polypeptides with non‐native conformations. The generated peptides translocate to the cytosol for complete degradation by the UPS. (d) Under oxidative stress conditions, folded IMS proteins are also retrotranslocated and degraded in the cytosol.

Work in yeast revealed that the ubiquitin‐proteasome system (UPS) constantly degrades a fraction of IMS precursors in the cytosol (Figure 3a), limiting the availability of precursors and acting as a negative regulator in protein biogenesis (Kowalski et al. 2018). Substrates that linger for too long in the cytosol after translation are ubiquitinated and degraded by the proteasome before reaching the mitochondria, avoiding their toxic accumulation in the cytosol (Bragoszewski et al. 2013).

Similarly, in human cells the dipeptidyl peptidases 8/9 (DPP8/9) mediate the N‐terminal cleavage of AK2 in the cytosol, before it reaches the IMS (Finger et al. 2020). This processing unmasks an IAP‐binding motif (IBM), which is ubiquitinated by the inhibitors of apoptosis (IAP) E3‐ligases, leading to proteasomal degradation of AK2. More than 100 mitochondrial proteins, including many IMS resident proteins, have a putative DPP8/9 recognition site which would unmask a potential IBM (Finger et al. 2020; Lapacz et al. 2025). This processing could be a potential mechanism counteracting the accumulation of slow importing IMS substrates as AK2.

Failure to degrade mitochondrial proteins and their accumulation in the cytosol would lead to mitochondrial precursor overaccumulation stress (mPOS) (Coyne and Chen 2018) and trigger cellular stress responses, such as the integrated stress response (ISR) (Mick et al. 2020) and the unfolded protein response activated by the mistargeting of proteins (UPRam) (Wrobel et al. 2015). These pathways aim to restore protein homeostasis by combining a reduction in mitochondrial protein load, inhibiting protein synthesis, and enhancing protein folding and degradation by increasing chaperones and proteases levels (refer to another review in this Special Issue for a more comprehensive discussion on this topic).

#### 4.1.2 During import

As extensively described above, the oxidative folding pathway acts as a QC mechanism ensuring the retention in the IMS only of correctly folded proteins. During the translocation through the TOM complex, IMS disulfide relay substrates form mixed disulfide bonds with CHCHD4/Mia40, potentially generating unproductive CHCHD4/Mia40‐substrate intermediates (Zarges and Riemer 2024). These intermediates are resolved by reducing machineries in the IMS, including thioredoxins and glutaredoxins. In yeast, Grx2 and Trx1 are partially localized in the IMS and regulate Mia40 redox balance (Kojer et al. 2015; Vögtle et al. 2012). The released substrate is then retrotranslocated to the cytosol, through the TOM complex, and degraded by the UPS (Figure 3b) (Habich et al. 2019a; Liao et al. 2025). Alternatively, stalled IMS bipartite precursor proteins are extracted from the OMM surface by the AAA protein ATAD1/Msp1 and degraded by the UPS via the mitochondrial compromised protein import response (mitoCPR) (Kim et al. 2024; Weidberg and Amon 2018).

#### 4.1.3 In the IMS

Protein surveillance does not end once proteins reach the IMS and fold into their native conformation. Fully imported proteins are constantly monitored by chaperones, such as CHCHD4/Mia40 and the small TIMM chaperones (TIMM8‐13), which contribute to mitochondrial protein import and ensure correct precursor folding and retention. Recent mammalian work also highlights a surveillance network of small heat shock proteins, such as HSPB1, that are targeted to the IMS upon proteotoxic stress and counteract protein aggregation (Adriaenssens et al. 2023). Additionally, disaggregases, such as CLPB/Skd3 and Hsp78, resolve protein aggregates and prevent their toxic accumulation, facilitating their refolding or degradation by mitochondrial proteases.

Folded proteins can be processed by IMS resident proteases, such as YME1L/Yme1, OMA1, and HTRA2 (Figure 3c). For example, the iAAA protease, the hexameric YME1L/Yme1 complex, cleaves the IMM fusion protein OPA1 and subsequently controls mitochondria dynamics (Anand et al. 2014). The iAAA protease also monitors the folding state of IMS proteins and degrades unfolded ones (Leonhard et al. 1999). Any polypeptide exposed to the IMS can be degraded by the iAAA protease, either from a soluble protein (Baker et al. 2012) or a membrane‐anchored protein in the OMM (Wu et al. 2018) or the IMM (Hsu et al. 2025). By controlling the turnover of small TIMM proteins and subunits of the TIM23 complex, YME1L also regulates mitochondrial protein import (Baker et al. 2012; Hsu et al. 2025). Another IMS protease, OMA1, surveils mitochondria proteostasis by cleaving stalled importing proteins in depolarized mitochondria (Krakowczyk et al. 2024). The peptides generated by the proteolytic activity of mitochondria proteases are then exported to the cytosol and trigger the mitochondrial unfolded protein response (UPRmt), therefore contributing to the general proteostasis cell response (Zhu et al. 2021) (refer to another review in this Special Issue for a more comprehensive discussion on this topic).

#### 4.1.4 Back to the cytosol

Not only the peptides generated by the mitochondrial proteases move from the IMS to the cytosol for complete degradation, but also entire proteins. Under oxidative stress conditions, mature proteins are reduced, lose their native conformation and retrotranslocate to the cytosol (Figure 3d) (Bragoszewski et al. 2015). These unfolded proteins cross the OMM through the Tom40 channel in an ATP‐dependent and membrane potential‐independent process (Bragoszewski et al. 2015; Liao et al. 2025). Finally, in the cytosol they are rapidly degraded by the proteasome, preventing their proteotoxic accumulation (Bragoszewski et al. 2015). The molecular mechanism mediating this process, and specifically the initial steps triggering substrates reduction and unfolding, has not been characterized yet and will require additional investigation. Although such mechanisms have been described only in yeast cells, we envision that higher eukaryotes must have a similar QC system to remove reduced IMS proteins under stress conditions. It is likely that they are retrotranslocated to the cytosol for degradation or are digested by mitochondrial proteases; however, further work is required to confirm these hypotheses.

### 4.2 IMS role in regulating mitochondrial proteome

#### 4.2.1 Proteins transiting in the IMS

Except for alpha‐helical OMM proteins, all nuclear‐encoded mitochondrial proteins transit in the IMS during their journey to their final location (Dimogkioka et al. 2021). Therefore, the IMS is a central hub for controlling the biogenesis of the organelle and avoiding accumulation of dysfunctional mitochondria (these pathways will be discussed more in detail in other reviews of this Special Issue).

In non‐photosynthetic eukaryotic organisms, beta‐barrel proteins are only localized in the OMM. They reach their final location by translocating into the IMS through the TOM complex; here they interact with the small TIMM chaperones, which ensure to keep them unfolded and soluble, and finally they are inserted into the OMM lipid bilayer by the SAM/TOB complex (Ganesan et al. 2024).

The small TIMM chaperones are also interacting with carrier proteins, once they have reached the IMS through the TOM complex. This allows maintaining the substrate in an import‐competent conformation until they are inserted into the IMM by the TIM22 complex (Dimogkioka et al. 2021).

Substrates with an MTS are targeted either to the matrix or the IMM via the TIM23 complex. In this case, the substrates are not directly exposed to the IMS since the TIM23 complex is in direct contact with the TOM complex, allowing the substrates to translocate the two membranes simultaneously in an unfolded state (Dimogkioka et al. 2021).

Overall, the IMS proteome directs mitochondrial proteins to their inter‐organelle location and ensures that they are kept in an import‐competent state during this process.

#### 4.2.2 IMS as an emergency storage compartment

Under import stress conditions, the IMS can act as an emergency storage compartment, preventing the accumulation of proteins that might have a deleterious effect in the cytosol.

Recently, it has been observed that, upon loss of the mitochondrial membrane potential, several matrix proteins cannot be imported and accumulate in the IMS. Specifically, mitochondrial ribosomal proteins (MRPs), known to have non‐canonical targeting sequences (Bykov et al. 2022), accumulate in the IMS of de‐energized mitochondria. It has been suggested that this novel mechanism, named mitochondrial triage of precursor proteins (MitoTraP), prevents association of unimported MRPs with assembly components of the cytosolic ribosome, which could interfere with cellular translation (Flohr et al. 2025). Mitochondrial ribosome subunits were also detected in the IMS of human cells upon treatment with uncouplers, suggesting that this process is conserved in higher eukaryotes (Kang et al. 2025). The details regulating this novel process are still not fully understood; it is unclear how MRPs translocate in the IMS and the structural features determining their trapping. The fate of MRP in the IMS is also unknown: are they degraded by mitochondria proteases or are they stored in the IMS and potentially re‐imported into the matrix when and if the membrane potential is restored?

Additionally, mitochondria have been proposed to act as an emergency storage compartment for cytosolic aggregation‐prone proteins in a process called mitochondria as guardian in cytosol (MAGIC) (Ruan et al. 2017; Wang et al. 2024). Under acute proteotoxic stress conditions, such as heat shock (Ruan et al. 2017) or glucose deprivation (Wang et al. 2024), some cytosolic proteins aggregate in yeast and associate with the mitochondrial surface. Here, they translocate into mitochondria relying on the disaggregase Hsp104, which unfolds them during the import process. Once in the mitochondrial matrix, the protease Pim1 degrades the aggregated proteins (Ruan et al. 2017). Few of these cytosolic proteins have been found also in the IMS, supporting the concept that this mitochondrial compartment contributes to counteract cellular proteotoxic stress (Ruan et al. 2017).

## 5 CONCLUSION

In summary, proteins are sorted to the IMS through numerous import pathways. A third of IMS proteins follow the MIA40 pathway, which is the most well‐characterized sorting mechanism but certainly is not the only way into the IMS. Particularly, the protein topologies targeted to the IMS, and specifically those accommodated by MIA40, are diverse and suggest this number will increase as proteomic and biochemical approaches improve. To ensure the IMS remains conducive for import, various QC mechanisms have evolved to maintain redox balance, prevent protein mislocalization, and proteotoxic accumulation. Thus, IMS import pathways and QC mechanisms cooperate to ensure proteostasis regulation in a concerted effort to maintain cellular homeostasis and mitochondrial function.

## AUTHOR CONTRIBUTIONS

**Fara van der Schans:** Conceptualization; writing – review and editing; data curation; writing – original draft. **Kostas Tokatlidis:** Funding acquisition; writing – review and editing; supervision. **Daniela G. Vitali:** Conceptualization; supervision; writing – review and editing; data curation; writing – original draft.

## FUNDING INFORMATION

Engineering and Physical Sciences Research Council, Grant Number UKRI141; Biotechnology and Biological Sciences Research Council, Grant Numbers: BB/T003804/1, BB/R009031/1, BB/X511948/1; James McCune Smith PhD studentship.

## CONFLICT OF INTEREST STATEMENT

The authors declare no conflicts of interest.

## Data Availability

Data sharing not applicable to this article as no datasets were generated or analysed during the current study.
